# Donor genetic and nongenetic factors affecting red blood cell transfusion effectiveness

**DOI:** 10.1172/jci.insight.152598

**Published:** 2022-01-11

**Authors:** Nareg H. Roubinian, Sarah E. Reese, Hannah Qiao, Colleen Plimier, Fang Fang, Grier P. Page, Ritchard G. Cable, Brian Custer, Mark T. Gladwin, Ruchika Goel, Bob Harris, Jeanne E. Hendrickson, Tamir Kanias, Steve Kleinman, Alan E. Mast, Steven R. Sloan, Bryan R. Spencer, Steven L. Spitalnik, Michael P. Busch, Eldad A. Hod, on behalf of the National Heart Lung and Blood Institute (NHLBI) Recipient Epidemiology and Donor Evaluation Study IV Pediatrics (REDS-IV-P)

**Affiliations:** 1 Division of Research, Kaiser Permanente Northern California, Oakland, California, USA.; 2 Vitalant Research Institute, San Francisco, California, USA.; 3 Westat, Rockville, Maryland, USA.; 4 Division of Biostatistics and Epidemiology, RTI International, Durham, North Carolina, USA.; 5 Division of Biostatistics and Epidemiology, RTI International, Atlanta, Georgia, USA.; 6 American Red Cross, Farmington, Connecticut, USA.; 7 Department of Medicine, University of Pittsburgh, Pittsburgh, Pennsylvania, USA.; 8 Department of Pathology, Johns Hopkins University, Baltimore, Maryland, USA.; 9 Department of Laboratory Medicine, Yale University, New Haven, Connecticut, USA.; 10 Vitalant Research Institute, Denver, Colorado, USA.; 11 Department of Pathology and Laboratory Medicine, University of British Columbia, Victoria, British Colombia, Canada.; 12 Versiti Blood Research Institute, Milwaukee, Wisconsin, USA.; 13 Department of Pathology, Children’s Hospital Boston, Boston, Massachusetts, USA.; 14 American Red Cross, Dedham, Massachusetts, USA.; 15 Department of Pathology and Cell Biology, Columbia University Irving Medical Center, New York, New York, USA.; 16 See Supplemental Acknowledgments for REDS-IV-P details.

**Keywords:** Hematology, Clinical practice, Genetic variation

## Abstract

**BACKGROUND:**

RBC transfusion effectiveness varies due to donor, component, and recipient factors. Prior studies identified characteristics associated with variation in hemoglobin increments following transfusion. We extended these observations, examining donor genetic and nongenetic factors affecting transfusion effectiveness.

**METHODS:**

This is a multicenter retrospective study of 46,705 patients and 102,043 evaluable RBC transfusions from 2013 to 2016 across 12 hospitals. Transfusion effectiveness was defined as hemoglobin, bilirubin, or creatinine increments following single RBC unit transfusion. Models incorporated a subset of donors with data on single nucleotide polymorphisms associated with osmotic and oxidative hemolysis in vitro. Mixed modeling accounting for repeated transfusion episodes identified predictors of transfusion effectiveness.

**RESULTS:**

Blood donor (sex, Rh status, fingerstick hemoglobin, smoking), component (storage duration, γ irradiation, leukoreduction, apheresis collection, storage solution), and recipient (sex, BMI, race and ethnicity, age) characteristics were associated with hemoglobin and bilirubin, but not creatinine, increments following RBC transfusions. Increased storage duration was associated with increased bilirubin and decreased hemoglobin increments, suggestive of in vivo hemolysis following transfusion. Donor *G6PD* deficiency and polymorphisms in *SEC14L4*, *HBA2*, and *MYO9B* genes were associated with decreased hemoglobin increments. Donor *G6PD* deficiency and polymorphisms in *SEC14L4* were associated with increased transfusion requirements in the subsequent 48 hours.

**CONCLUSION:**

Donor genetic and other factors, such as RBC storage duration, affect transfusion effectiveness as defined by decreased hemoglobin or increased bilirubin increments. Addressing these factors will provide a precision medicine approach to improve patient outcomes, particularly for chronically transfused RBC recipients, who would most benefit from more effective transfusion products.

**FUNDING:**

Funding was provided by HHSN 75N92019D00032, HHSN 75N92019D00034, 75N92019D00035, HHSN 75N92019D00036, and HHSN 75N92019D00037; R01HL126130; and the National Institute of Child Health and Human Development (NICHD).

## Graphical abstract

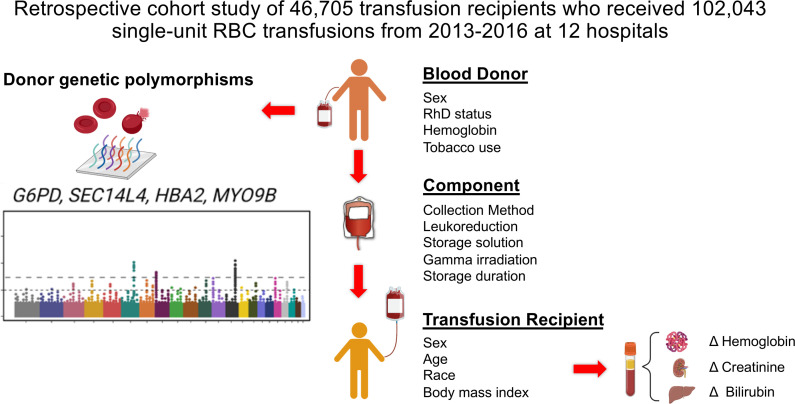

## Introduction

RBC transfusions can be a critical life-saving intervention for hospitalized and chronically transfused patients. In contrast to pharmaceutical-grade drugs, there is substantial variability in hemoglobin dose and RBC quality among units of blood (1, 2). Blood donors are a heterogenous population for which biologic, genetic, and behavioral variables interact with various manufacturing and storage conditions to contribute to variation in the hemoglobin dose of packed RBC units and their responses to cold storage. For logistical reasons, RBCs are refrigerator stored for up to 6 weeks in the United States, resulting in altered RBC biology (i.e., the storage lesion) (3, 4). A subset of storage-damaged RBCs is cleared from the circulation after transfusion, releasing large amounts of hemoglobin, which is primarily catabolized into iron and bilirubin (2, 5, 6). Finally, intravascular hemolysis may affect kidney function and exacerbate other human diseases (7, 8). Thus, because these laboratory values are often measured in hospitalized patients requiring transfusion, we assessed transfusion effectiveness by the change in hemoglobin, bilirubin, or creatinine levels following transfusion.

Despite substantial intersubject variability in how RBCs tolerate refrigerated storage, there is considerable intrasubject reproducibility in storage quality (i.e., some donors are “good storers,” whereas others are “poor storers”) (2, 9–12). It is increasingly recognized that blood donor demographics, social behaviors, and genetic factors play a role in stored blood product quality. A key focus of transfusion medicine research is to understand the role of donor genetics in observed RBC storage variability (4, 13). For example, glucose-6-phosphate dehydrogenase (*G6PD*) deficiency, an X-linked disorder affecting the ability of RBCs to handle oxidant stress, is the most common human enzymopathy, affecting approximately 400 million people worldwide (14, 15). Interestingly, blood donor *G6PD* deficiency increases the susceptibility of RBCs to the storage lesion, resulting in decreased posttransfusion recovery of transfused RBCs (12). More recently, several other potentially novel and known genetic polymorphisms were found to be associated with measures of osmotic and oxidative hemolysis in vitro, and they are predicted to affect transfusion effectiveness (16). However, translating findings from in vitro studies of blood donors and component manufacturing practices to clinically relevant outcomes in transfused patients is challenging (17, 18). Thus, in this retrospective study, we utilized a large, linked vein-to-vein database to identify predictors of transfusion effectiveness following single RBC unit transfusions. In a cohort of approximately 50,000 evaluable patients across 12 academic and community hospitals participating in the NHLBI-funded REDS-III program, we examined associations between donor, component, and recipient characteristics (including donor genetic polymorphisms) and RBC transfusion effectiveness.

## Results

### Number of transfusion events and demographics.

**Figure 1:**
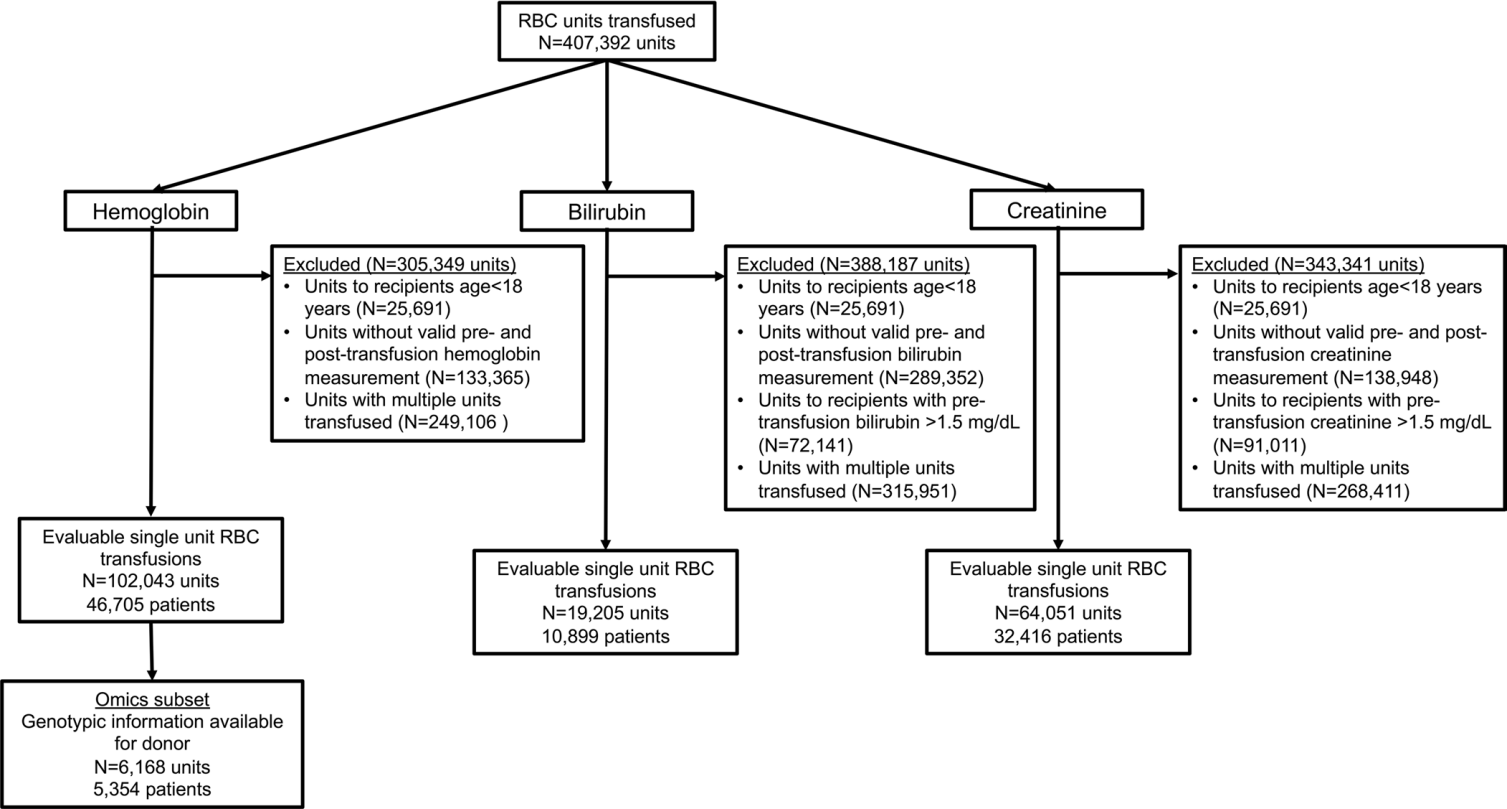
Study flow diagram.

We identified 46,705 and 5354 patients, with 102,043 and 6168 evaluable single RBC unit transfusion episodes, in the full cohort and the RBC-Omics Study subset (in which donor genetic polymorphism information was available) (19), respectively; this allowed for examining the hemoglobin increment as an outcome (Figure 1). In addition, there were 10,899 patients with 19,205 single RBC unit transfusion episodes and 32,416 patients with 64,051 single RBC unit transfusion episodes in whom bilirubin and creatinine increments were examined, respectively. The RBC-Omics Study subset was similar to the full cohort except for race and ethnicity, because non-White donors were preferentially recruited in the REDS-III RBC-Omics Study (e.g., mean of 2.8% Black donors in the full cohort and 6.8% in the RBC-Omics subset; Table 1). Furthermore, RBC units transfused from the RBC-Omics subset were more likely to be from whole blood rather than apheresis donations as compared with the full cohort (91.2% versus 76.6% by whole blood). Recipients with bilirubin measures available were more likely than those with hemoglobin measures available to receive irradiated RBCs (52.2% versus 24.2%), concomitant plasma (6.5% versus 4.0%), and platelets (13.0% versus 5.9%). Finally, recipients with creatinine measures available were very similar to those with hemoglobin measures (Table 2).

### Bivariate associations confirm factors associated with hemoglobin and bilirubin increment.

The mean ± SD pretransfusion hemoglobin, bilirubin, and creatinine levels were 7.35 ± 0.84 g/dL, 0.67 ± 0.34 mg/dL, and 0.86 ± 0.29 mg/dL and were measured at 4 (IQR, 2–8), 8 (IQR, 4–15), and 6 (IQR, 3–10) hours before transfusion, respectively (Table 2). The posttransfusion hemoglobin, bilirubin, and creatinine levels were measured at 21 (IQR, 18–25), 15 (IQR, 8–19), and 67 (IQR, 61–72) hours after transfusion, respectively. The mean ± SD hemoglobin, bilirubin, and creatinine increments were 0.96 ± 0.84 g/dL, 0.19 ± 0.44 mg/dL, and –0.01 ± 0.29 mg/dL, respectively. The timing and hemoglobin levels in the RBC-Omics study subset closely paralleled those of the full hemoglobin cohort. The bivariate analyses of blood donor, component, and recipient characteristics (Supplemental Table 1; supplemental material available online with this article; https://doi.org/10.1172/jci.insight.152598DS1) were mostly consistent with prior studies (1), with recipient characteristics such as sex and BMI having strong associations with hemoglobin increments. Donor race and ethnicity were not associated with hemoglobin or bilirubin increments. Significant associations were consistent between the full hemoglobin cohort and the RBC-Omics study subset.

Because it is expected that prolonged refrigerator storage prior to transfusion is associated with an approximately 10% decrease in posttransfusion RBC recovery on average (9), we hypothesized that storage duration would decrease the hemoglobin increment; and indeed, interpolation from the parameter estimates of the bivariate analysis (Supplemental Table 1) confirmed an 8% decrease in hemoglobin increment by the end of the 6-week allowable storage duration. Furthermore, prior studies suggest that extravascular hemolysis following clearance of the storage-damaged RBCs would result in increased circulating bilirubin levels (2). Bivariate analysis supported this finding with an approximately 0.03 mg/dL observed increase in bilirubin increment per week of storage (Supplemental Table 1). No donor factors were associated with the change in creatinine following transfusion. In addition, the parameter estimates for component and recipient factors associated with the creatinine change following transfusion were small and clinically insignificant. Sensitivity analyses examining relative rather than absolute changes in creatinine levels after transfusion yielded similar findings (results not shown). Thus, we concluded that single RBC transfusions have a limited effect on creatinine levels, and multivariable models exploring change in creatinine as an outcome are not reported.

### Multivariable analysis suggests that increased storage duration is associated with hemolysis.

**Figure 2:**
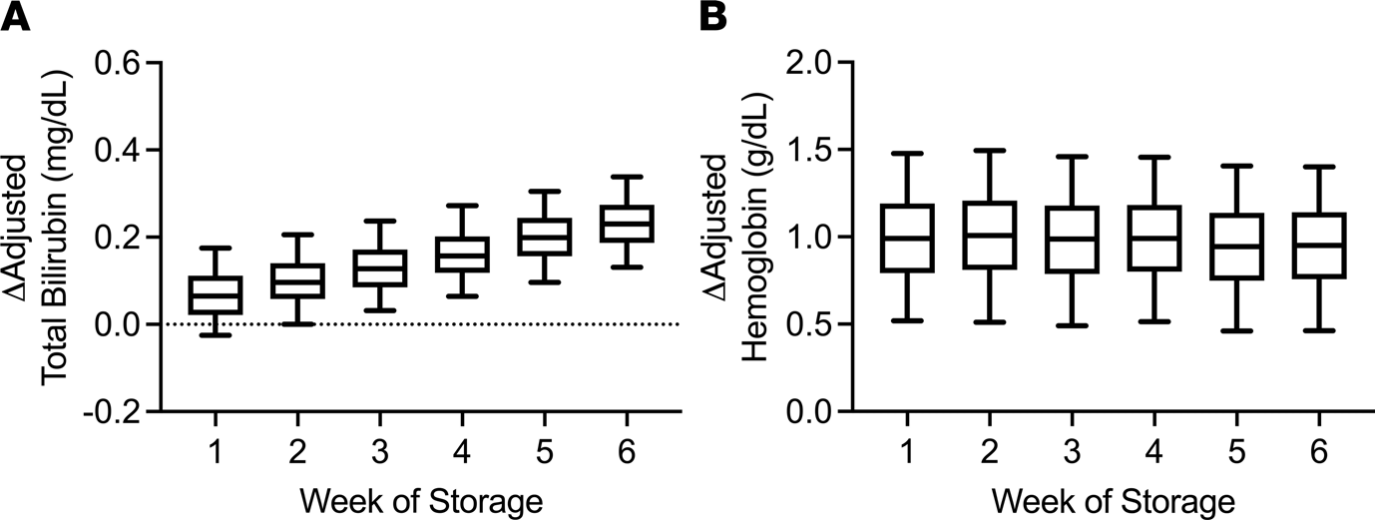
Effect of storage age and irradiation on hemoglobin and bilirubin increments. (**A** and **B**) The adjusted changes in total bilirubin (*n* = 19,205) and hemoglobin (*n* = 102,043) levels between pre- and posttransfusion laboratory measures are shown stratified by week of storage, as labeled. Hemoglobin and bilirubin increments were adjusted for concomitant donor, component, and recipient factors presented in Table 3 and plotted as derivatives of the regression equation. *P* < 0.0001 for linear association between storage duration and change in laboratory measure for both figure panels.

On multivariable analysis, donor factors predicted to increase the amount of hemoglobin in a unit (e.g., male donor, increased donor hemoglobin, tobacco use; Table 3) (1, 20) were associated with both increased hemoglobin and bilirubin increments. Furthermore, component characteristics associated with decreased amounts of hemoglobin in a unit (e.g., apheresis collection and leukoreduction) (21, 22) were associated with both decreased hemoglobin and bilirubin increments. In contrast, longer RBC storage duration was associated with decreased hemoglobin increment but increased bilirubin increment (Figure 2), suggesting that storage-damaged RBCs are cleared from the circulation and that hemoglobin derived from these lysed RBCs is catabolized to bilirubin. In addition, there was a significant interaction term between storage duration and irradiation such that irradiation was associated with a negative effect on hemoglobin increment after 19 days of storage. Finally, recipient factors associated with increased blood volume (e.g., male and BMI) were associated with a decreased hemoglobin increment. Other recipient demographic factors (e.g., age, race and ethnicity, receipt of concomitant platelets or plasma, issue location) were associated with both hemoglobin and bilirubin increments.

### RBC units from donors with G6PD deficiency are associated with decreased hemoglobin increment.

**Figure 3:**
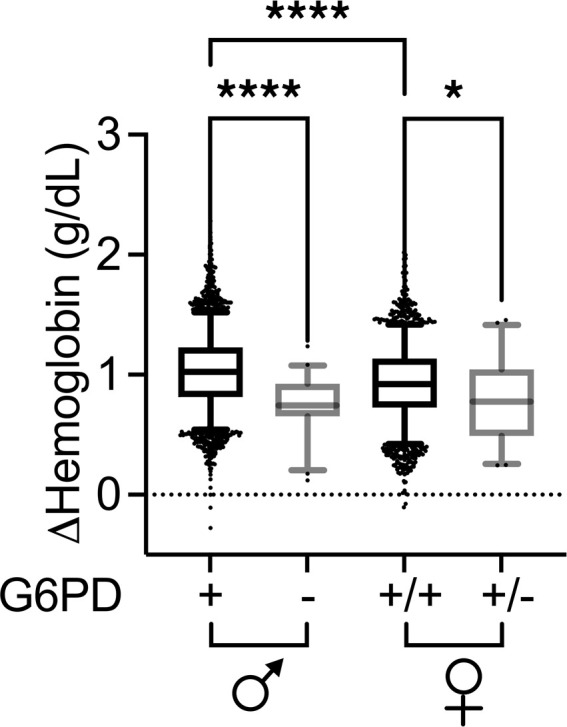
Adjusted hemoglobin increments stratified by sex and glucose-6-phosphate dehydrogenase (G6PD) deficiency. Adjusted change in hemoglobin level between pre- and posttransfusion measures is shown stratified by male (*n* = 3458) and female (*n* = 2584) sex and *G6PD* genotype, as labeled. As *G6PD* is X-linked, males labeled (–) are G6PD deficient (*n* = 43) and females labeled (+/–) are heterozygous (*n* = 46). Hemoglobin is adjusted for concomitant donor, component, and recipient factors. ****P < 0.0001, *P < 0.05 for 1-way ANOVA comparison as indicated. Line represents median, boxes represent 25th to 75th percentile, and whiskers represent the 5th to 95th percentile.

Given that a subset of the blood donors in our linked database had associated genotype data from the REDS-III RBC-Omics Study (Figure 1), we examined single nucleotide polymorphisms (SNPs) identified in that study that were associated with oxidative and osmotic hemolysis of stored RBCs in vitro (16). The first candidate SNP explored was in the *G6PD* gene. A prior study suggests that, based on current FDA criteria, RBCs from G6PD-deficient donors would not meet the requirements for suitable storage quality and are associated with a 6.8% reduction in posttransfusion recovery by the end of the allowable storage period, as compared with RBCs from donors with the WT *G6PD* allele (12). Thus, to validate the models, we examined *G6PD* deficiency in the linked database with respect to hemoglobin increment. Compared with the WT allele (*n* = 3458), donor *G6PD* deficiency variants (*n* = 43) in male donors were strongly associated with a decreased mean ± SD hemoglobin increment in transfused recipients (1.02 ± 0.30 g/dL versus 0.76 ± 0.23 g/dL, *P* < 0.0001; Figure 3) in our multivariable model accounting for other donor, component, and recipient factors. In parallel to prior studies (1), RBC units from *G6PD*-normal female donors exhibited a lower hemoglobin increment than *G6PD*-normal males (0.93 ± 0.30 g/dL versus 1.02 ± 0.30 g/dL, *P* < 0.0001; Figure 3). Smaller hemoglobin increments associated with RBC units from female donors heterozygous for *G6PD* deficiency alleles (*n* = 46) compared with those with WT alleles (*n* = 2584) were also statistically significant (0.81 ± 0.35 g/dL versus 0.93 ± 0.30 g/dL, *P* = 0.04; Figure 3). No relatively rare female homozygous G6PD-deficient donors were enrolled in the RBC-Omics study. Bilirubin changes were not analyzed due to the insufficient number of transfusion episodes in the bilirubin model when incorporating only units from donors participating in the RBC-Omics Study. Finally, relative to RBC units from WT allele donors, RBC units from G6PD-deficient donors were associated with increased incidence (OR, 2.35 [95% CI, 1.16–4.76]; *P* = 0.017; *n* = 43) and number of additional RBC transfusions (1.14 [95% CI, 0.75–1.52] versus 0.69 [95% CI, 0.65–0.73] RBC units; *P* = 0.03) within 48 hours of the index transfusion event.

### Other donor SNPs are associated with hemoglobin increments in recipients.

We explored the effects of other donor SNPs on transfusion effectiveness, as defined by the recipient hemoglobin increment (bilirubin increment models were not considered due to sample size limitations), in our multivariable model adjusting for donor, component, and recipient factors. We first compared allele frequency in all RBC-Omics donors with that of donors represented in our linked database to test whether there was selection bias (Supplemental Table 2); the relative prevalence of minor alleles was similar for the SNPs in all RBC-Omics donors with the relative prevalence of minor alleles present in single RBC unit transfusion episodes. Of the 22 genes associated with osmotic or oxidative hemolysis in the RBC-Omics study (Supplemental Table 3), those that were significantly associated with the hemoglobin increment in our study were *SEC14L4*, *MYO9B*, and *HBA2* (Table 4). Although mutations in *G6PD* and *HBA2* are associated with anemia and human disease (15, 23), *SEC14L4* and *MYO9B* are not previously known to affect RBC function. Relative to RBC units from homozygous dominant donors, RBC units from homozygous recessive donors were associated with increased incidence in the receipt of additional RBC transfusions within 48 hours of the index transfusion event for *SEC14L4* (OR, 1.54 [95% CI, 1.03–2.30]; *P* = 0.035; *n* = 247), but not for *HBA2* (OR, 2.46 [95% CI, 0.40–15.2]; *P* = 0.34; *n* = 10) or *MYO9B* (OR, 1.13 [95% CI, 0.94–1.35]; *P* = 0.20; *n* = 1,113). In parallel, the number of additional RBC transfusions for recipients of index units from homozygous recessive donors compared with homozygous dominant donors was increased for *SEC14L4* (0.95 [95% CI, 0.72–1.17] versus 0.70 [95% CI, 0.64–0.75] RBC units; *P* = 0.03), but not for *HBA2* (1.12 [95% CI, –0.15–2.40] versus 0.65 [95% CI, 0.61–0.69] RBC units; *P* = 0.46) or *MYO9B* (0.60 [95% CI, 0.51–0.70] versus 0.62 [95% CI, 0.55–0.69] RBC units; *P* = 0.76).

Recognizing that blood component modifications have been associated with increased rates of hemolysis (1, 24, 25), we additionally examined the association of unit irradiation in the subset of SNPs associated with in vitro hemolysis. Of those, 4 genes were associated with significant decrements in hemoglobin levels for the homozygous recessive allele for only irradiated units (Supplemental Table 4). Among irradiated units, recessive alleles for *SEC14L4* again correlated with reduced hemoglobin increments, as did SNPs for *Hexokinase 1* (*HK1*), for which mutations are associated with a nonspherocytic hemolytic anemia (26), and *Aquaporin 1* (*AQP1*), an integral membrane protein regulating RBC volume in response to tonicity (27). Given the relative frequency of the homozygous recessive alleles for *SEC14L4* and *MYO9B* in blood donors and transfused units, we examined our primary model covariates stratifying by SNP allele for each of these 2 genes and found that donor, component, and patient characteristics were similar for recipients of single-unit RBC transfusions, thereby supporting a true effect of these gene variants rather than confounding (Supplemental Table 5, A and B).

## Discussion

We examined the role of blood donor, component, and recipient characteristics, including donor genetic polymorphisms, on 3 laboratory parameters that we defined as measures of RBC transfusion effectiveness. In this large, linked, “vein-to-vein” database, we first examined previously described donor, component, and recipient factors associated with increments in hemoglobin (1, 20) and bilirubin (2, 11) to establish the validity of our models and to determine covariates that should be included in the final multivariable model. Validated by other studies (1, 2, 11, 20), hemoglobin and bilirubin increments correlated positively with factors associated with increased RBC unit mass. In contrast, prolonged RBC storage was associated with increased bilirubin and reduced hemoglobin increments in transfused patients, findings indicative of hemolysis (1, 2, 11). We did not observe any clinically significant associations in the creatinine increment following transfusion, suggesting that variability in transfused RBC products has limited impact on this gross measure of kidney function. We next sought to examine potentially novel associations between donor SNPs associated with in vitro hemolysis measures on our outcomes of transfusion effectiveness. Given prior evidence supporting a negative effect of G6PD deficiency on posttransfusion recovery (12) and transfusion effectiveness in patients with sickle cell disease (28), we first examined G6PD deficiency in our more limited RBC-Omics Study cohort. Transfusion of single RBC units from donors with G6PD deficiency not only decreased the hemoglobin increment, but it was also associated with an increased need for subsequent transfusion in the ensuing 48 hours. Thus, this represents the first demonstration that transfusion of blood from donors with G6PD deficiency has a clinical impact. We extended these results to additional SNPs and discovered that polymorphisms in the *SEC14L4*, *MYO9B,* and *HBA2* genes are associated with a reduced hemoglobin increment. The potential clinical relevance of these findings is highlighted by the significant increase in the need for additional RBC transfusions within 48 hours of the index transfusion event for single-unit RBC transfusions from donors with a polymorphism in *SEC14L4*. Thus, these findings may ultimately lead to improved precision patient blood management approaches for decreasing RBC transfusions in vulnerable patient populations.

Allogeneic RBC units are complex biological products prepared from donated blood with significant variability in hemoglobin dose and quality. In parallel to other recent studies, we identified changes in the hemoglobin increment related to individual blood donor, component manufacturing, and recipient factors (1, 29). Some of these differences are due to differences in the RBC mass of the collected blood. For example, males have higher hemoglobin levels than females; in this study, both male sex and higher donor hemoglobin levels at collection were associated with a larger hemoglobin increment. In contrast, manufacturing practices such as apheresis collection and leukoreduction are associated with a smaller hemoglobin increment due to reductions in overall RBC mass collected (for apheresis RBC) or due to loss of RBC during component processing (due to leukoreduction) (21, 22). Although some donor and component characteristics individually played small roles in hemoglobin increments, they remained relevant to the variation observed in clinical practice even after multivariable adjustment. For example, an irradiated unit of RBCs from a male donor with G6PD deficiency stored for 42 days would be associated with a median hemoglobin increment that is 0.51 g/dL lower than a nonirradiated unit from a male donor without G6PD deficiency transfused on the first day of storage. Assuming an average single-unit transfusion is associated with a 1 g/dL increase in hemoglobin, this represents an approximately 50% decrease in the effective dose of circulating hemoglobin.

Although not previously demonstrated, findings of both increased bilirubin and hemoglobin increments (e.g., with transfusion of male-derived or whole blood RBC units) are not unexpected and likely represent clearance of a larger mass of damaged RBCs in units with higher doses of hemoglobin. RBC storage duration was the exception to the aforementioned correlation, with prolonged storage being strongly associated with a reduced hemoglobin and an increased bilirubin increment. This particular relationship was previously demonstrated in healthy volunteers transfused with autologous RBC units (2). Although several randomized controlled trials (30–34) did not observe significant improvements in outcomes of patient who received fresher RBC transfusions, it is increasingly recognized that, due to significant donor variability, the chronological storage age of RBC units does not necessarily indicate the metabolic age of the RBCs transfused (35). This is the first retrospective study to our knowledge to demonstrate an effect of storage age on posttransfusion bilirubin levels, and this approach may be useful for identifying patient populations and clinical outcomes that may be affected by hemolysis from transfused RBCs. Indeed, because increases in bilirubin correlate with other markers of hemolysis, such as serum iron and nontransferrin bound iron (2), these findings may be particularly relevant for chronically transfused patients for whom overall iron burden results in harm (36–38).

The REDS-III RBC-Omics Study identified multiple potentially novel donor genetic polymorphisms associated with hemolysis in vitro after prolonged storage (16). Many of the SNPs identified were in genes associated with hemoglobinopathies, enzymopathies, and membranopathies, all of which could plausibly affect RBC recovery following storage and transfusion. Due to sample size limitations, we focused our analyses on the hemoglobin increment as the main outcome measure for the genetic variants identified in the RBC-Omics Study. Using multivariable analyses, we found a smaller hemoglobin increment in 2 of the 5 genome-wide significant loci for oxidative hemolysis: *G6PD* and *SEC14L4*. Out of 18 loci for osmotic hemolysis examined for decreased hemoglobin increment, we observed 2 significant associations: *HBA2* and *MYO9B*. Although SNPs in *G6PD* and *HBA2* are associated with human disease, clinical phenotypes for *SEC14L4* and *MYO9B* are not yet described. A sensitivity analysis of relevant donor, component, and patient characteristics did not identify any obvious differences between the alleles of these 2 SNPs (Supplemental Table 5, A and B). Interestingly, and requiring further validation, transfusion of RBC units from *G6PD* heterozygous female donors was associated with a decreased hemoglobin increment, suggesting that female heterozygotes may have an intermediary phenotype for oxidative hemolysis both in vitro and in vivo. In parallel with decreased hemoglobin increments, we found an increase in the likelihood and number of subsequent transfusions in patients receiving a single RBC unit from donors with polymorphisms in *G6PD* or *SEC14L4*. Although other clinical consequences of these SNPs are unclear and will require additional mechanistic investigations, it is notable that the above 4 SNPs were among 7 genetic variants that correlated with in vivo hemolysis in a study in sickle cell patients (16).

Additional factors may further influence the impact of donor genetic polymorphisms associated with oxidative or osmotic hemolysis. Several studies found that manufacturing method, γ irradiation, and storage solution influence hemolysis rates. For example, γ irradiation increases plasma free hemoglobin in the supernatant of RBC units by disrupting RBC membrane integrity, which results in decreased RBC recovery following transfusion, particularly after longer storage durations (24, 25, 39–42). Component modifications may further increase hemolysis in RBC units from donors with particular genetic variants. Thus, we hypothesized that donor genetic polymorphisms and component factors may have “multi-hit” interactions on RBC quality. In this setting, the first “hit” is a particular donor genetic polymorphism, and the second “hit” is γ irradiation (Supplemental Table 4). Because recessive alleles for *G6PD* or *HBA2* are relatively infrequent in this donor population, these could not be examined robustly. However, several SNPs, including the homozygous recessive allele for *SEC14L4*, were associated with a decreased hemoglobin increment following transfusion of irradiated RBC units. Although speculative, a cumulative effect of donor genetics and component modifications on RBC quality align with the proposed concept of the “metabolic age” of RBCs (35), which may vary significantly from changes expected with chronological storage age alone.

The current study has several limitations. The retrospective and observational nature of the study design limits the ability to infer causation for the factors associated with decreased transfusion effectiveness. Nonetheless, the models confirmed previous prospective, randomized study findings of decreased hemoglobin, and increased bilirubin increments associated with increased storage duration (2). Furthermore, RBCs from donors with G6PD deficiency were associated with decreased posttransfusion recovery in prospective studies (12, 28, 43). Thus, our statistical models were able to validate these prior associations, and this lends support to the validity of the analytic approach. However, given the number of variables examined, the possibility of a chance result (Type I error) for any given association exists, and each association will require further validation. In particular, given the lack of adjustment for a FDR and multiple comparisons, the SNPs in *SEC14L4*, *MYO9B*, and *HBA2* require further confirmation, as their effects on transfusion effectiveness have yet to be explored in other studies, to our knowledge. In addition, subjects with evaluable before-and-after transfusion bilirubin levels were clearly different from those with just evaluable hemoglobin levels (e.g., they were more likely to receive irradiated blood products and concomitant plasma and/or platelet units). These subjects were also more likely to be recipients of multiunit RBC episodes where bleeding and additional i.v. fluids may have impacted our outcomes. In parallel with other characteristics, uncommon SNPs are also less likely to impact hemoglobin increment when occurring in multiunit transfusion events; nevertheless, they contribute to the significant variation in hemoglobin increments observed in clinical practice in conjunction with other donor and component factors. Future studies of multiunit transfusion events are needed in a controlled setting, including study of the role of platelet and plasma transfusions to demonstrate the generalizability of our findings. Although we limited analysis to pretransfusion bilirubin levels below 1.5 mg/dL to mitigate the role of patient conditions (e.g., liver disease) from affecting the results, our findings may still be prone to unmeasured confounding. However, sensitivity analyses examining relative changes in creatinine levels and various pretransfusion thresholds for our outcomes did not yield different results. Due to sample size limitations, our analysis was underpowered to identify associations of uncommon donor alleles that had small effect sizes, nor were we able to examine the impact of donor genetics on posttransfusion bilirubin increments. To this end, the NIH-funded REDS-IV-P program is planning a prospective trial in chronically transfused patients with sickle cell disease, thalassemia, or pediatric cancer to confirm and expand upon the genetic determinants of transfusion effectiveness (RBC-Improving Transfusions for Chronically Transfused Recipients [RBC-IMPACT Study]). Finally, despite the associations of donor, component, and recipient variables with decreased transfusion effectiveness, as defined by a decreased hemoglobin increment or an increased bilirubin increment demonstrated by this study, we did not explore whether decreased transfusion effectiveness is associated with patient harm other than an increased need for subsequent transfusion. Our current analyses were limited to single-unit episodes, and potential harmful effects may be greater when accounting for cumulative transfusion exposures. A recent study found that a limited set of donor and manufacturing characteristics correlated with smaller hemoglobin increments, which were then associated with additional RBC transfusion requirements in the patients (20). We similarly observed an increased odds of requiring a subsequent transfusion in patients transfused a single RBC unit from a donor with polymorphisms in *G6PD* or *SEC14L4*. Nonetheless, we believe that future studies focusing on specific patient populations (e.g., chronically transfused patients with hemoglobinopathies) and specific outcome measures (e.g., iron overload) can better address the issue of potential harm.

In conclusion, we found significant associations between blood donor (e.g., sex, Rh status, fingerstick hemoglobin, tobacco use), component (e.g., storage duration, γ irradiation, leukoreduction, apheresis collection, storage solution), and recipient (e.g., sex, race and ethnicity, BMI, age) characteristics, including specific donor genetic polymorphisms in 4 genes (*G6PD*, *SEC14L4*, *HBA2*, and *MYO9B*), and effectiveness measures following single-unit RBC transfusion events. The strong associations between increased storage duration and donor G6PD deficiency on transfusion effectiveness support the validity of this approach for identifying factors that influence transfusion effectiveness. These findings highlight the power of large, linked “vein-to-vein” databases that link blood donor and recipient outcome information. Furthermore, the current models expand upon previous models examining variables affecting the hemoglobin increment (1) and are the first to our knowledge to describe the impact of donor, component, and recipient factors on bilirubin increment following transfusion, a laboratory measure of hemolysis in vivo. These findings support the concept that donor factors, including genetic variables, can modulate RBC stability in storage and could provide relevant donor selection criteria for improving transfusion outcomes in selected populations. For example, many blood donors are already genotyped, and phenotypically matched blood is targeted to select populations to prevent alloimmunization (44). In a similar fashion, a precision transfusion medicine approach may benefit chronically transfused patients, who may be at increased risk of harm from transfusion of RBCs from donors with *G6PD* deficiency or other SNPs (28, 45). Thus, identifying genetically susceptible RBC units — which may result in suboptimal transfusion responses — or, conversely, RBC units that better tolerate cold storage could provide a strategy more advanced than the current approach using a random distribution of donors and RBC products irrespective of donor genotype or short- and long-term transfusion requirements of the recipient.

## Methods

### General study design.

We conducted a retrospective cohort study using electronic health records from the NHLBI REDS-III program available as public use data through BioLINCC (46, 47). The database included blood donor, component, and recipient data collected at 12 academic and community hospitals from 4 geographically dispersed regions in the United States (Connecticut, Pennsylvania, Wisconsin, and California) for the 4-year period from January 1, 2013, to December 31, 2016. Genotype data from the subset of blood donors who participated in the REDS-III RBC-Omics study (19) were included as part of the linked dataset.

### Study population and definitions.

Data regarding blood donor demographics (e.g., age, sex, race and ethnicity, ABO/Rh), collection date and methods (e.g., whole blood or apheresis), as well as component manufacturing characteristics (e.g., timing of leukoreduction, additive solution, irradiation status), were extracted for each RBC unit collected. Available donor genetic polymorphism data from the REDS-III RBC-Omics study (19) was linked to issued RBC units using donor identification numbers. Among transfusion recipients, we included all adult patients who received a single RBC unit during 1 or more transfusion episodes between January 1, 2013, and December 30, 2016. Recipient details included age, sex, race and ethnicity, and BMI, along with storage age and blood product issue date, time, and location of transfused RBCs. Information on concomitant transfusion of other blood components (e.g., platelets, plasma) during the RBC transfusion episode was included. We collected hemoglobin, creatinine, and bilirubin levels measured by the corresponding clinical laboratory prior to and following each RBC transfusion event.

### Transfusion exposures and outcome measures.

All single RBC unit transfusion episodes were included in this analysis. A RBC unit transfusion episode was defined as any single RBC transfusion from a single donor with both informative pre- and posttransfusion laboratory measures and without any other RBC units transfused in the intervening period between lab draws. The 3 outcome measures of interest were hemoglobin, total bilirubin, and creatinine increments following a single RBC unit transfusion episode. These outcomes were defined as the difference between the posttransfusion and pretransfusion levels. Pretransfusion thresholds for these measures were chosen to limit patient confounding (e.g., underlying hepatic or renal disease). For pretransfusion hemoglobin, the value used was the most proximal hemoglobin measurements prior to RBC transfusion — but, at most, 24 hours prior to transfusion. For posttransfusion hemoglobin (g/dL), the laboratory measure nearest to 24 hours after transfusion, but between 12 and 36 hours following transfusion, was based on prior studies. For pretransfusion bilirubin (mg/dL), the most proximal total bilirubin measurement prior to transfusion — but, at most, 7 days prior to transfusion — was used. Furthermore, to exclude patients with preexisting liver disease, the pretransfusion bilirubin had to be below 1.5 mg/dL for the transfusion to be considered. For posttransfusion bilirubin, the most proximal laboratory measure to the transfusion — but, at most, 24 hours after transfusion — was used based on clinical trial data (2, 11, 32). For pretransfusion creatinine (mg/dL), the most proximal measurement prior to the transfusion time — but, at most, 24 hours prior to transfusion — was used. Furthermore, to exclude patients with preexisting kidney disease, the pretransfusion creatinine had to be below 1.5 mg/dL for the transfusion to be considered. Finally, to assess for changes in kidney function, the maximum creatinine level most proximal to 72 hours after transfusion — but, at most, 96 hours after transfusion — was derived from acute kidney injury criteria (48).

### Statistics.

We assessed the univariable association of all a priori–selected donor, component, and recipient covariates with the outcomes using linear regression. Any covariate that was associated (α = 0.2) with the outcome was included in a multivariable linear mixed effects model accounting for repeated transfusion recipients. Additional a priori selected interaction terms were included in the multivariable models based on hypotheses of modifiers of clinical outcomes (e.g., γ irradiation × storage duration). Cook’s distance was used to detect outliers (Cook’s distance threshold < 0.1 was used to detect outliers) and stepwise model selection on the fixed effects (α = 0.05) was used to select the final full covariate model for each outcome. Initial models were fit on the subset of the data that had no missing observations for all covariates. Once the final model covariates were selected, the model including just those covariates was reassessed on the larger data set, excluding observations with missing values for only the selected covariates to maximize sample size. We tested for multicollinearity in the final models using generalized variance inflation factor values adjusted for the number of regressors, and we performed additional outlier assessment. Fixed marginal effects from multivariable regression models were used to correlate delta hemoglobin and bilirubin levels by week of storage duration. For the subset of recipients of RBC units from REDS-III RBC-Omics donors (19), we examined alleles of donor genetic polymorphisms associated with measures of osmotic or oxidative hemolysis in vitro. Accounting for the same donor, component, and recipient factors, multivariable linear regression assessed associations between alleles of donor SNPs and the posttransfusion hemoglobin increment. For donor SNPs associated with the posttransfusion hemoglobin increment, we further examined the incidence and number of subsequent RBC transfusions occurring within 48 hours of the index transfusion event using multivariable logistic regression. Sample size estimates for different allele frequencies and effect sizes were calculated (Supplemental Table 6). Two-sided *P* values less than 0.05 were considered to be statistically significant. Analyses were performed in R 4.0.3 (R Core Team, 2020) (49, 50) and Stata Version 14.1 (StataCorp). R package lme4 was used to fit linear mixed effects models (49).

### Study approval.

Approval for data collection had been obtained from the IRB at each participating institution prior to submission as public use data to BioLINCC.

## Author contributions

NHR, SR, RC, BC, RG, SK, AM, SLS, BS, SRS MPB, and EAH conceived of and designed the study. NHR, SR, HQ, CP, FF, GP, BC, TK, SK, AM, BS, MPB, and EAH acquired and prepared the data. NHR, SR, CP, FF, GP, and EAH performed statistical analyses. NHR, SR, FF, GP, RC, BC, MG, RG, BH, JH, TK, SK, AM, SLS, BS, SRS, MPB, and EAH interpreted data. NHR, SR, and EAH wrote the manuscript. All authors contributed to the critical revision of the manuscript for important intellectual content.

## Supplementary Material

Supplemental data

ICMJE disclosure forms

## Figures and Tables

**Table 4:**
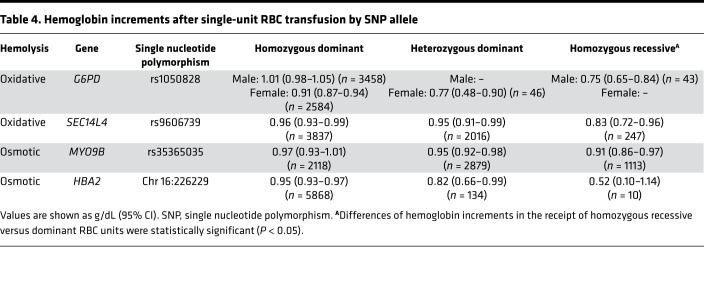
Hemoglobin increments after single-unit RBC transfusion by SNP allele

**Table 3:**
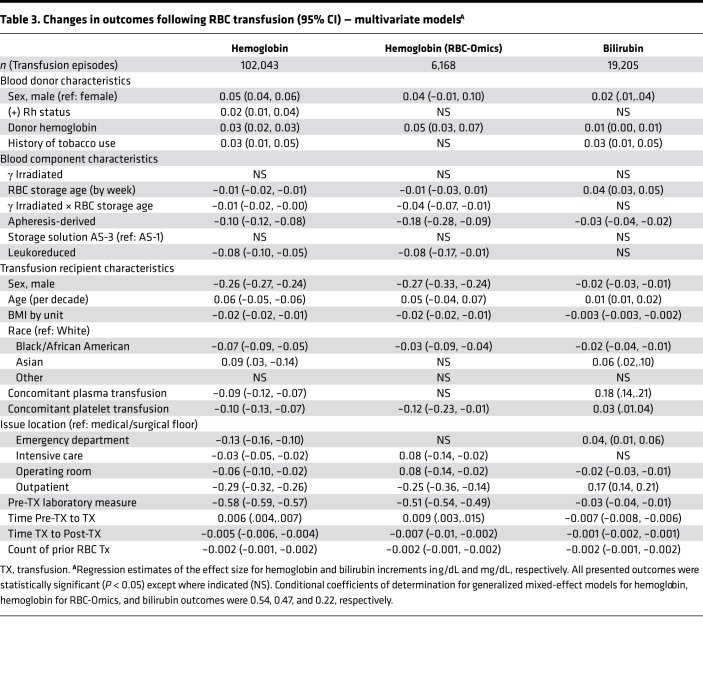
Changes in outcomes following RBC transfusion (95% CI) — multivariate models^A^

**Table 2:**
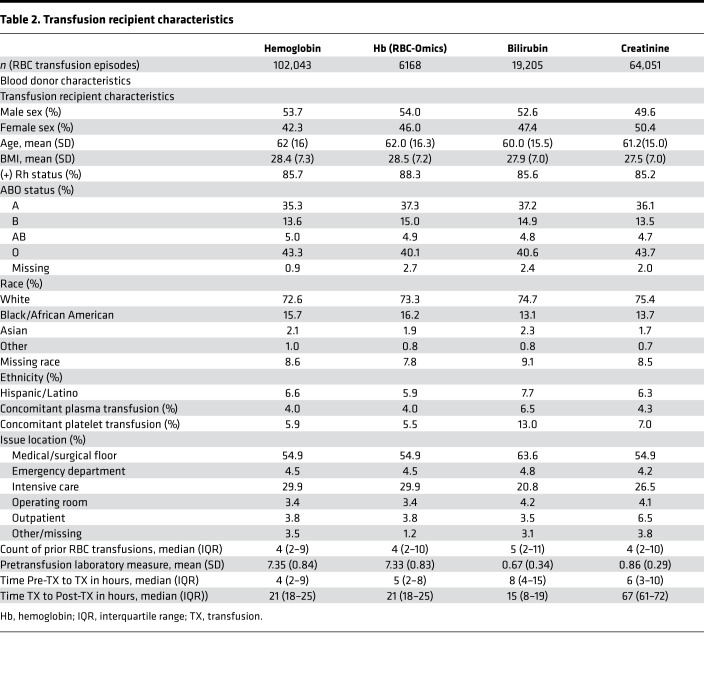
Transfusion recipient characteristics

**Table 1:**
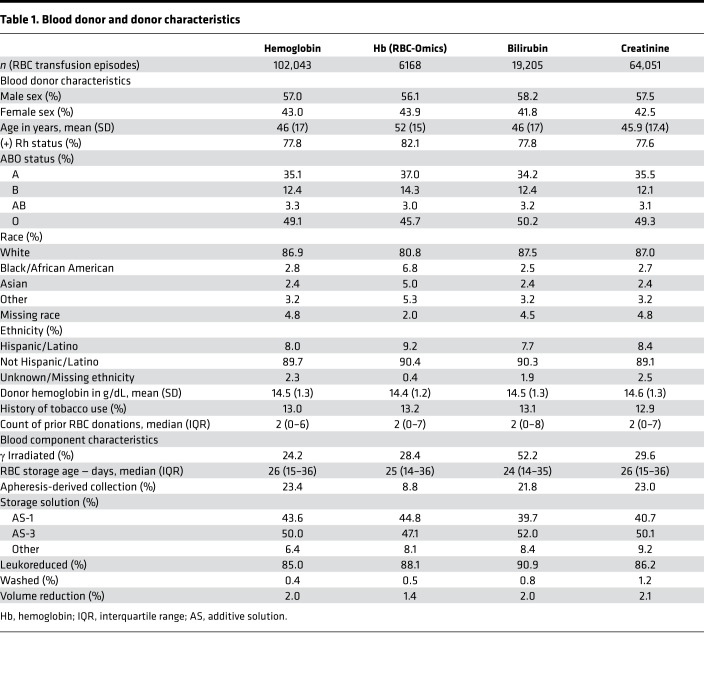
Blood donor and donor characteristics

## Version 1 11/18/2021

In-Press Preview

## Version 2 01/11/2022

Electronic publication
