# Supplementary material for: Donor genetic and nongenetic factors affecting red blood cell transfusion effectiveness
Source: JCI Insight. 2022 Jan 11;7(1):e152598. doi: 10.1172/jci.insight.152598 (PMC8765041; doi:10.1172/jci.insight.152598)
Supplement: Supplemental data [file jciinsight-7-152598-s161.pdf]

**Supplementary Table 1: Univariable hemoglobin, bilirubin and creatinine increments after RBC transfusion**

| | Hemoglobin | Hb (RBC-Omics) | Bilirubin | Creatinine |
|---|---|---|---|---|
| N (RBC transfusion episodes) | 102,043 | 6,168 | 19,205 | 64,051 |
| **Blood Donor Characteristics** | | | | |
| Sex, male (ref: female) | 0.07 (0.06, .08) | 0.05 (0.00, .10) | 0.03 (.01, 0.4) | NS |
| Age in years by decade | -0.01 (-.01, -.00) | NS | NS | NS |
| (+) Rh status | 0.03 (-.02, .05) | NS | -0.01 (-.03, -0.01) | NS |
| ABO, O (vs. all others) | NS | NS | -0.03 (-.04, -0.02) | NS |
| Donor hemoglobin level | 0.03 (.02, .04) | 0.06 (.04, .08) | 0.01 (.01, .02) | NS |
| History of tobacco use | 0.04 (.02, .07) | NS | 0.03 (.01, .05) | NS |
| Race (ref: White) | | | | |
| Black/African American | NS | NS | NS | NS |
| Asian | NS | NS | 0.05 (.02, .09) | NS |
| Other | 0.04 (.00, .08) | NS | NS | NS |
| Unknown | NS | NS | 0.05 (.03, .08) | NS |
| **Blood Component Characteristics** | | | | |
| Gamma irradiation | 0.05 (.03, .06) | NS | -0.08 (-.09, -.07) | 0.02 (.01, .02) |
| RBC storage age in days | -0.01 (-.01, -.00) | -0.02 (-.03, -0.00) | 0.03 (.02, .03) | -0.00 |
| Apheresis-derived | -0.06 (-.08, -.04) | -0.14 (-.25, -.03) | NS | NS |
| Storage solution, AS-3 (ref: AS-1) | 0.09 (.08, .11) | 0.06 (.00, .11) | 0.06 (.00, .11) | NS |
| Leukoreduction | -0.13 (-.15, -.11) | -0.08 (-.16, .00) | -0.08 (-.16, .00) | 0.02 (.01, .03) |
| **Transfusion Recipient Characteristics** | | | | |

| | | | | |
|---|---|---|---|---|
| Sex, Male | -0.27 (-.28, -.26) | -0.30 (-.28, -.25) | -0.02 (-.03, -.01) | -0.00 |
| Age (per decade) | 0.03 (.02, .03) | 0.02 (.00, .04) | 0.01 (.01, .02) | 0.00 |
| Body mass index (per unit) | -0.02 (-.02, -.01) | -0.02 (-.02, -.01) | -0.00 | 0.00 |
| (+) Rh status | 0.04 (.02,.06) | NS | NS | NS |
| ABO, O (vs. all others) | NS | NS | -0.03 (-.04, -.02) | NS |
| Race (ref: White) | | | | |
| Black/African American | 0.10 (.08, .12) | 0.14 (.06, .21) | -0.02 (-.04, -.00) | 0.01 (.00, .02) |
| Asian | 0.24 (.20, .29) | 0.31 (0.12, .51) | 0.16 (.12, .19) | 0.02 (.00, .03) |
| Other | NS | NS | NS | NS |
| Concomitant plasma | -0.51 (-.55, -.48) | -0.27 (-.40, -.13) | 0.41 (.38, .45) | 0.05 (.04, .06) |
| Concomitant platelets | -0.34 (-.37, -.32) | -0.24 (-.35, -.12) | 0.03 (.01, .05) | 0.04 (.03, .05) |
| Issue location (ref: ward) | | | | |
| Emergency department | NS | NS | 0.10 (.08, .13) | -0.05 (.07, .04) |
| Intensive care | -0.11 (-.13, -.10) | -0.15 (-.21, -.09) | 0.03 (.02, .05) | 0.03 (.02, .03) |
| Operating room | -0.28 (-.32, -.24) | -0.38 (-.52, -.24) | -0.03 (-.06, -.00) | NS |
| Outpatient | NS | -0.97 (-1.09, -.85) | 0.37 (.34, .40) | NS |
| Pre-TX laboratory measure | -0.53 (-.53, -.52) | -0.50 (-.52, -.48) | .04 (.03, .06) | -0.09 (-.10, -.08) |
| Time Pre-TX to TX (per hour) | -0.005 (-.006, -.003) | -0.004 (-.01,-.002) | NS | 0.00 |
| Time TX to Post-TX (per hour) | -0.008 (-.009,-.007) | -0.008 (-.01, -.003) | -0.01 (-.01, -.01) | 0.00 |
| Count of prior RBC Tx | -0.002 (-.003,-.002) | -0.002 (-.004,-.000) | -0.003 (-.003, -.002) | 0.00 |

RBC=red blood cell; Hb=hemoglobin; TX = transfusion
Linear model estimates and 95% confidence intervals.
All presented outcomes were statistically significant ($p<0.05$) except where indicated (NS)

| Supplementary Table 2: Mean allele frequency for single nucleotide polymorphisms associated with hemolysis in genotyped blood donors and single RBC unit transfusion events from genotyped blood donors | | | | | | | | | |
|---|---|---|---|---|---|---|---|---|---|
| | | | | Genotyped blood donor; n (%) | | | Single RBC unit transfusions from genotyped blood donor; n (%) | | |
| **chromosome** | **Nearest gene** | **Hemolysis** | **Single nucleotide polymorphism** | **Homozygous Major** | **Heterozygous** | **Homozygous Minor** | **Homozygous Major** | **Heterozygous** | **Homozygous Minor** |
| **1** | *SPTA1* | Osmotic | rs857725 | 6,875 (54.4) | 4,729 (37.4) | 1044 (8.3) | 3428 (55.6) | 2276 (36.9) | 459 (7.5) |
| **1** | - | Oxidative | rs17661899 | 9353 (74.7) | 2865 (22.9) | 305 (2.4) | 4363 (71.6) | 1571 (25.8) | 163 (2.7) |
| **1** | *TFB2M* | Osmotic | rs35558093 | 7579 (60.3) | 4272 (34.0) | 710 (5.7) | 3492 (57.1) | 2274 (37.2) | 349 (5.7) |
| **2** | *MFSD2B* | Osmotic | rs55707417 | 10073 (79.6) | 2423 (19.2) | 156 (1.2) | 4868 (78.9) | 1218 (19.8) | 81 (1.3) |
| **5** | *GLRX* | Oxidative | rs6871404 | 7009 (55.5) | 4775 (37.8) | 853 (6.8) | 3387 (55.1) | 2316 (37.7) | 444 (7.2) |
| **6** | *EYS* | Osmotic | rs78484557 | 12040 (95.6) | 540 (4.3) | 8 (0.1) | 5791 (94.4) | 337 (5.5) | 5 (0.1) |
| **7** | *AQP1* | Osmotic | rs1859838 | 8642 (68.3) | 3485 (27.5) | 532 (4.2) | 4209 (68.3) | 1741 (28.3) | 213 (3.5) |
| **7** | *IKZF1* | Osmotic | rs12718598 | 3414 (27.0) | 6114 (48.4) | 3100 (24.5) | 1613 (26.3) | 2967 (48.3) | 1564 (25.5) |
| **8** | *ANK1* | Osmotic | rs4737009 | 6319 (49.8) | 5028 (39.6) | 1339 (10.6) | 3251 (52.6) | 2411 (39.0) | 524 (8.5) |
| **9** | *MIR4289* | Osmotic | Chr 9:91408347 | 11507 (91.6) | 1024 (8.2) | 32 (0.2) | 5519 (90.2) | 589 (9.6) | 13 (0.2) |
| **10** | *HK1* | Osmotic | rs16926246 | 9958 (78.4) | 2567 (20.2) | 170 (1.3) | 4755 (76.9) | 1340 (21.7) | 90 (1.5) |
| **11** | *HBB* | Osmotic | rs3345248 | 12367 (99.2) | 97 (0.8) | 2 (0) | 6044 (99.6) | 22 (0.4) | 0 (0) |
| **11** | *SWAP70* | Osmotic | rs93139 | 3898 (30.7) | 6176 (48.7) | 2608 (20.6) | 2030 (32.8) | 2985 (48.3) | 1168 (18.0) |
| **11** | *ARHGAP42* | Osmotic | rs11606890 | 9961 (79.0) | 2423 (19.2) | 224 (1.8) | 4866 (79.2) | 1178 (19.2) | 101 (1.6) |
| **12** | *ALDH2* | Osmotic | rs4646777 | 8577 (67.8) | 3676 (29.0) | 402 (3.2) | 4173 (67.8) | 1792 (29.1) | 191 (3.1) |
| **13** | - | Osmotic | rs118149920 | 11946 (96.1) | 471 (3.8) | 10 (0.1) | 5910 (96.9) | 182 (3.0) | 5 (0.1) |
| **16** | *HBA2* | Osmotic | Chr 16:226229 | 11975 (96.6) | 403 (3.2) | 21 (0.2) | 5924 (97.6) | 140 (2.3) | 10 (0.1) |
| **16** | *PIEZO1* | Osmotic | rs837763 | 3956 (31.2) | 6231 (49.2) | 2476 (19.6) | 1930 (31.3) | 3058 (49.5) | 1185 (19.2) |
| **17** | *SLC4A1* | Osmotic | rs7222349 | 4034 (31.9) | 5460 (43.1) | 3172 (25.0) | 2159 (34.9) | 2737 (44.3) | 1285 (20.8) |
| **19** | *GPX4* | Oxidative | rs8178962 | 3806 (32.4) | 5622 (48.0) | 2293 (19.6) | 1852 (32.0) | 2843 (49.1) | 1092 (18.9) |
| **19** | *MYO9B* | Osmotic | rs35365035 | 4388 (34.7) | 5948 (47.0) | 2321 (18.3) | 2118 (34.7) | 2879 (47.1) | 1113 (18.2) |
| **22** | *SEC14L4* | Oxidative | rs9606739 | 8280 (65.5) | 3845 (30.4) | 510 (4.0) | 3837 (62.9) | 2016 (33.1) | 247 (4.0) |
| **23** | *G6PD* | Oxidative | rs1050828 | 12421 (97.8) | 169 (1.3) | 104 (0.8) | 6042 (98.5) | 46 (0.8) | 43 (0.7) |

| Supplementary Table 3: Hemoglobin increments after single unit RBC transfusion by SNP allele – g/dL [95% Confidence Interval] | | | | | |
|---|---|---|---|---|---|
| **Hemolysis** | **Single nucleotide polymorphism** | **Gene** | **Homozygous dominant** | **Heterozygous dominant** | **Homozygous recessive** |
| Osmotic | rs857725 | *SPTA1* | 0.95 [0.92-0.98] | 0.98 [0.95-1.02] | 0.96 [0.87-1.04] |
| Oxidative | rs17661899 | - | 0.96 [0.93-0.99] | 0.97 [0.93-1.02] | 0.93 [0.78-1.07] |
| Osmotic | rs35558093 | *TFB2M* | 0.97 [0.94-1.00] | 0.95 [0.91-0.99] | 1.00 [0.90-1.10] |
| Osmotic | rs55707417 | *MFSD2B* | 0.95 [0.93-0.98] | 0.99 [0.94-1.05] | 0.99 [0.79-1.19] |
| Oxidative | rs6871404 | *GLRX* | 0.95 [0.92-0.98] | 0.99 [0.94-1.02] | 0.93 [0.84-1.02] |
| Osmotic | rs78484557 | *EYS* | 0.96 [0.94-0.98] | 1.01 [0.91-1.12] | 0.98 [0.21-1.76] |
| Osmotic | rs1859838 | *AQP1* | 0.96 [0.93-0.99] | 0.97 [0.92-1.01] | 0.98 [0.86-1.11] |
| Osmotic | rs12718598 | *IKZF1* | 0.97 [0.92-1.01] | 0.97 [0.93-1.00] | 0.95 [0.91-1.00] |
| Osmotic | rs4737009 | *ANK1* | 0.94 [0.90-0.97] | 0.99 [0.95-1.03] | 1.00 [0.92-1.08] |
| Osmotic | Chr 9:91408347 | *MIR4289* | 0.97 [0.94-0.99] | 0.92 [0.85-1.00] | 1.00 [0.51-1.49] |
| Osmotic | rs16926246 | *HK1* | 0.94 [0.90-0.97] | 0.99 [0.95-1.03] | 0.87 [0.68-1.06] |
| Osmotic | rs3345248 | *HBB* | 0.96 [0.94-0.99] | 0.90 [0.52-1.27] | - |
| Osmotic | rs93139 | *SWAP70* | 0.98 [0.94-1.02] | 0.95 [0.92-0.99] | 0.95 [0.89-1.00] |
| Osmotic | rs11606890 | *ARHGAP42* | 0.97 [0.95-1.00] | 0.92 [0.87-0.98] | 0.97 [0.79-1.16] |
| Osmotic | rs4646777 | *ALDH2* | 0.95 [0.93-0.98] | 0.98 [0.93-1.02] | 1.04 [0.91-1.17] |
| Osmotic | rs118149920 | - | 0.96 [0.94-098] | 0.97 [0.83-1.10] | 1.13 [0.23-2.02] |
| Osmotic | Chr 16:226229* | *HBA2* | 0.96 [0.94-0.98] | 0.84 [0.68-1.00] | 0.53 [0.11-1.16] |
| Osmotic | rs837763 | *PIEZO1* | 0.99 [0.94-1.03] | 0.95 [0.92-0.98] | 0.96 [0.91-1.01] |
| Osmotic | rs7222349 | *SLC4A1* | 0.96 [0.92-1.00] | 0.96 [0.92-0.99] | 0.97 [0.92-1.02] |
| Oxidative | rs8178962 | *GPX4* | 0.94 [0.90-0.98] | 0.97 [0.94-1.01] | 0.95 [0.89-1.01] |
| Osmotic | rs35365035* | *MYO9B* | 0.98 [0.94-1.02] | 0.96 [0.93-1.00] | 0.92 [0.87-0.98] |
| Oxidative | rs9606739* | *SEC14L4* | 0.97 [0.94-1.00] | 0.96 [0.92-1.00] | 0.84 [0.72-0.96] |
| Oxidative | rs1050828* | *G6PD* | 0.97 [0.94-0.99] | 0.85 [0.58-1.13] | 0.70 [0.43-0.96] |

RBC=red blood cell; SNP=single nucleotide polymorphism; CI=confidence interval
* Outcomes statistically significant ($p<0.05$)

| **Supplementary Table 4. Hemoglobin increments after single unit RBC transfusion by SNP allele & irradiation status – g/dL [95% Confidence interval]** | | | | | |
|---|---|---|---|---|---|
| **Gene** | **Single nucleotide polymorphism** | **Modification** | **Homozygous dominant** | **Heterozygous dominant** | **Homozygous recessive** |
| ***SEC14L4*** | rs9606739 | Unirradiated | 1.01 [0.97-1.04] | 0.97 [0.93-1.02] | 0.93 [0.79-1.06] |
| | | Irradiated | 0.88 [0.82-0.93] | 0.93 [0.86-1.01] | 0.72 [0.52-0.93] |
| ***HK1*** | rs16926246 | Unirradiated | 0.99 [0.96-1.03] | 0.99 [0.93-1.05] | 0.97 [0.73-1.21] |
| | | Irradiated | 0.90 [0.85-0.95] | 0.86 [0.77-0.95] | 0.67 [0.37-0.98] |
| ***No Gene*** | rs176618991 | Unirradiated | 0.99 [0.96-1.02] | 1.00 [0.95-1.06] | 1.02 [0.85-1.20] |
| | | Irradiated | 0.89 [0.84-0.94] | 0.90 [0.81-0.98] | 0.71 [0.45-0.97] |
| ***AQP1*** | rs1859838 | Unirradiated | 0.93 [0.90-0.96] | 0.92 [0.88-0.97] | 1.01 [0.88-1.15] |
| | | Irradiated | 0.83 [0.78-0.88] | 0.84 [0.76-0.92] | 0.74 [0.50-0.98] |

**Supplementary Table 5a: Donor, component, & patient characteristics for transfusion recipients of single RBC units by *SECL414* alleles**

| N (episodes) | Homozygous dominant (n=3,837) | Heterozygote (n=2,016) | Homozygous recessive (n=247) |
|---|---|---|---|
| **Blood Donor Characteristics** | | | |
| Sex (% male) | 56 | 58 | 61 |
| Age in years (median, (IQR)) | 56 (43-63) | 56 (45-64) | 57 (42-65) |
| Hemoglobin level (mean, (SD)) | 14.3 (1.2) | 14.4 (1.1) | 14.4 (1.3) |
| **Blood Component Characteristics** | | | |
| Apheresis-derived (%) | 6 | 7 | 4 |
| Gamma irradiation (%) | 27 | 27 | 27 |
| RBC storage age -days (median (IQR)) | 27 (16-36) | 27 (16-36) | 27 (15-36) |
| **Transfusion Recipient Characteristics** | | | |
| Sex (% male) | 54 | 54 | 53 |
| Age (median, (IQR)) | 64 (56-74) | 64 (52-74) | 64 (54-72) |
| Body mass index (median, (IQR)) | 28 (24-33) | 28 (24-33) | 27 (23-31) |
| Pre-transfusion Hb in g/dL (mean, (SD)) | 7.5 (1.0) | 7.5 (1.0) | 7.5 (0.8) |

**Supplementary Table 5b: Donor, component, & patient characteristics for transfusion recipients of single RBC units by *MYO9B* alleles**

| N (episodes) | Homozygous dominant (n=2,365) | Heterozygote (n=3,163) | Homozygous recessive (n=1,219) |
|---|---|---|---|
| **Blood Donor Characteristics** | | | |
| Sex (% male) | 59 | 55 | 58 |
| Age in years (median, (IQR)) | 56 (43-64) | 56 (44-64) | 54 (43-62) |
| Hemoglobin level (mean, (SD)) | 14.4 (1.2) | 14.3 (1.1) | 14.4 (1.2) |
| **Blood Component Characteristics** | | | |
| Apheresis-derived (%) | 6 | 6 | 6 |
| Irradiation (%) | 26 | 27 | 27 |
| RBC storage age -days (median, (IQR)) | 27 (15-36) | 27 (16-36) | 27 (17-36) |
| **Transfusion Recipient Characteristics** | | | |
| Sex (% male) | 55 | 53 | 52 |
| Age (median, (IQR)) | 64 (53-75) | 63 (53-73) | 64 (53-74) |
| Body mass index (median, (IQR)) | 28 (24-33) | 28 (24-33) | 27 (23-33) |
| Pre-transfusion Hb in g/dL (mean, (SD)) | 7.5 (1.0) | 7.5 (1.0) | 7.5 (0.9) |

**Supplementary Table 6: Sample size estimates for SNP allele frequency and estimated effect size**

| ***Power*** | **Allele Frequency** | **Effect Size** | **N (∆Hemoglobin; *mu* = 1.0, SD = 0.9)** | **N (∆Bilirubin; *mu* = 0.0, SD = 0.4)** |
|---|---|---|---|---|
| *80%* | 50% | 0.05 | 5,082 | 1,001 |
| | | 0.10 | 1,268 | 247 |
| | | 0.15 | 561 | 108 |
| | | 0.20 | 314 | 59 |
| | 20% | 0.05 | 7,943 | 1,566 |
| | | 0.10 | 1,983 | 389 |
| | | 0.15 | 879 | 170 |
| | | 0.20 | 493 | 94 |
| | 10% | 0.05 | 14,124 | 2,787 |
| | | 0.10 | 3,528 | 694 |
| | | 0.15 | 1,566 | 306 |
| | | 0.20 | 879 | 170 |
| | 1% | 0.05 | 128,432 | 25,366 |
| | | 0.10 | 32,105 | 6,339 |
| | | 0.15 | 14,267 | 2,815 |
| | | 0.20 | 8,023 | 1,582 |

SNP=single nucleotide polymorphism

**The NHLBI REDS-IV-P domestic program is the responsibility of the following persons:**

Hubs: A.E. Mast and J.L. Gottschall, Versiti Wisconsin, Milwaukee, Wisconsin, USA. E.A. Hod, Columbia University Medical Center, New York, New York, USA, and B.S. Sachais, New York Blood Center, New York, New York, USA. B.S. Custer, Vitalant Research Institute, San Francisco, California, USA, and E.P. Vichinsky, Benioff Children's Hospital Oakland, Oakland, California, USA. J.E. Hendrickson, Yale University School of Medicine, New Haven, Connecticut, USA, and B.R. Spencer, American Red Cross, Dedham, Massachusetts, USA. Data coordinating center: S.M. Mathew and D.R. Harris, Westat, Rockville, Maryland, USA. N.L. Luban, Children's National Medical Center, Washington, DC. Central laboratory: M.P. Busch and P.J. Norris, Vitalant Research Institute, San Francisco, California, USA. Publications Committee Chairman: P.M. Ness, Johns Hopkins University, Baltimore, Maryland, USA. Steering Committee Chairpersons: S.H. Kleinman, University of British Columbia, Victoria, BC, Canada C.D. Josephson, Emory University, Atlanta, Georgia, USA. National Institute of Child Health and Human Development (NICHD): R. Tamburro: National Heart, Lung, and Blood Institute, National Institutes of Health: S.A. Glynn and K. Malkin
